# Short- and Long-Term Performance of Pipe Compounds Containing Polyethylene Post-Consumer Recyclates from Packaging Waste

**DOI:** 10.3390/polym14081581

**Published:** 2022-04-13

**Authors:** Paul J. Freudenthaler, Joerg Fischer, Yi Liu, Reinhold W. Lang

**Affiliations:** 1 Institute of Polymeric Materials and Testing, Johannes Kepler University Linz, Altenberger Straße 69, 4040 Linz, Austria; joerg.fischer@jku.at (J.F.); reinhold.lang@jku.at (R.W.L.); 2 Borealis Polyolefine GmbH, Innovation Headquarters, St. Peterstraße 25, 4021 Linz, Austria; yi.liu@borealisgroup.com

**Keywords:** plastics recycling, pipe materials, slow crack growth, cracked round bar, polyethylene, post-consumer, recyclate

## Abstract

The polymer industry is pushed to present solutions that lead to a circular plastics economy. High plastic packaging waste recycling targets will eventually lead to a high availability of packaging material recyclates. Although the use of polyethylene terephthalate (PET) recyclates is prescribed by regulations to be used in new PET bottles, no such regulation prescribes the use of polyethylene recyclate (rPE) in new products. One possibility of using rPE, which is considered by the European Union, is the use within pipe materials. Pipe applications demand a certain property profile, most prominently a high slow crack growth (SCG) resistance, which is not met by most packaging materials or recyclates made from it. Hence, this work investigates the use of commercially available post-consumer recyclates out of high-density polyethylene from packaging applications in compounds together with high SCG-resistant virgin PE pipe material with a PE100-RC specification. Two rPEs were acquired from German producers and blended to compounds consisting of 25 m%, 50 m% and 75 m% recyclate. These compounds, together with the pure recyclates and several virgin pipe materials acting as benchmarks were tested in terms of short- and long-term mechanical performance and with other basic characterization methods. Several compounds exceeded the performance of one tested virgin PE pipe material, an injection molding PE80 grade, in several categories. The content of recyclate needed to outperform this benchmark grade was mostly dependent on the resulting melt flow rate (MFR) of the compound and thus also of the MFR of the pure recyclate. Furthermore, different levels of polypropylene contaminations within the recyclates resulted in differently contaminated compounds. This is proved to influence the SCG resistance too, as compounds of similar MFRs but with different SCG resistances were found.

## 1. Introduction

Regulations and recent marketing activities have pushed the plastic industry to present solutions that will lead to a circular plastics economy. In the European Union (EU), the European Commission aids this development by declaring goals for higher use of recycled plastics in new products [1] and recycling targets for plastic packaging waste [2]. Although there are distinctive percentage minima for the recyclate content in polyethylene terephthalate (PET) beverage bottles [3], no regulations exist for the use of polyethylene (PE) post-consumer recyclate (PCR), or rather recycled polyethylene (rPE) from packaging waste.

Nevertheless, rPE is used and advertised within (non-food) packaging products. Furthermore, the European commission also investigates the use of PCRs in non-packaging applications such as pipes, as they show “good potential for uptake of recycled content” [1].

The use of post-consumer polyethylene pipe recyclates is rare and the usefulness questionable. Given the long use-cycle of pipes, post-use polyethylene pipe waste material might be several decades old with inferior performance to modern pipe material formulations. Although several European standards allow the use of non-virgin material originating from pipe products in accordance with European pipe standards, they, at least for now, distinctly forbid the use of recyclates from non-pipe products or external sources [4,5,6,7,8]. Apart from the origin of the material, performance characteristics dependent on the application (from high-pressure gas pipes to non-pressure drainage pipes) are limiting factors. The restricted usage of recyclates from waste sources other than pipes is plausible for high-performance and high-risk piping applications such as pressurized gas or drinking-water pipes. Nevertheless, rPE might still be used to (partly) replace virgin material within non-pressure low-risk piping applications such as drainage, sewerage, or jacket pipes, as long they fulfill the necessary performance characteristics.

Pipe resins must undergo an extensive testing program before being admitted for use within pipe products. They must, depending on the pipe application, comply with property limits concerning the density, oxidation induction time, melt flow rate, water content, carbon black content, and finally carbon black and/or pigment dispersion [6]. Many of these properties depend on the final processing step, i.e., after final additivation and compounding of the resin.

However, a distinctive feature of pipe resins is their mechanical performance, which is also more challenging to achieve. Therefore, as a first step of material development, these properties are worthwhile to investigate. This usually includes tests on produced pipes to measure properties such as the resistance to rapid crack propagation via the S4 test [9] or the resistance to slow crack growth (SCG) via the notched pipe test [10]. Although these tests, without question, are the most realistic simulation of a pipe’s mechanical performance, they are too costly and time-consuming in the early stages of material development and for the initial screening of many potential pipe resins. For the testing of the compounds, in terms of material development, other tests are suggested within standards such as the strain hardening modulus test [11], the full notch creep test [12], and the cracked round bar (CRB) test [13].

Preceding experiments show a successful incorporation of rPE from different product categories with virgin PE100 grade into compounds by fulfillment of several short- and long-term performance parameters [14,15,16]. Juan et al. [14] found out that blending rPE with a virgin pipe material was able to improve some short-term performance characteristics such as yield stress, flexural modulus, or rapid crack propagation resistance, which was measured via plane stress impact energy, compared to the virgin material. However, in their findings, the SCG resistance steadily decreased with a higher recyclate content for each used recyclate category. Therefore, the SCG resistance presents an important indicator for how much of a certain rPE can be compounded with a virgin pipe grade before the resulting compound fails to meet performance requirements.

In the present paper, two commercially available post-consumer rPEs coming from plastics packaging waste were blended with PE100-RC, a PE100 pipe grade with raised SCG resistance [6], and subsequently characterized in terms of short- and long-term mechanical performance to determine their applicability for lower-performance piping applications. Several virgin pipe grades of different pressure classes were tested in terms of SCG resistance and used as benchmarks to evaluate the performance of the produced compounds. Within this paper, the CRB test method [13] was used for investigation of the SCG resistance, as it is asked for by pipe standards [6,7], broadly accepted by the scientific community [17,18,19,20,21], and the authors have experience with this method as shown in previous publications [22,23,24]. Furthermore, the mechanical tests were supplemented by basic characterizations to allow for a comprehensive property profile.

However, the most promising candidates from these first steps of material development must be submitted to more tests on the resins and on pipe level to fulfill the requirements necessary for admission as a pipe resin, which is not within the scope of this study.

## 2. Materials and Methods

The purest available rPE in each case from two German recycling companies were provided for the purpose of this research. These two recyclates will henceforth be called rPE-A and rPE-B. rPE-A was delivered as gray pellets and mostly consisted of pre-sorted household plastic waste. rPE-B was delivered as natural-colored pellets and mostly consisted of post-consumer packaging (e.g., shampoo and detergent bottles) from the “yellow-bag”, which is a separate collection stream for plastic packaging products in Germany [25].

Virgin PE pipe-grade materials with PE100-RC, PE100 and PE80 specifications were acquired for comparison and compounding in the form of pellets. Although PE100-RC and PE100 were extrusion-grade materials with a specified melt flow rate (MFR) of 0.25 g/10 min and represent the upper performance benchmarks, the PE80 grade with its advertised MFR of 0.8 g/10 min is intended to be used, e.g., for the injection molding of pipe fittings, and represents the lower performance benchmark.

Blends of virgin PE100-RC and recyclates were compounded on an Leistritz ZSE MAXX 18 40/48D twin-screw extruder (Leistritz Extrusionstechnik GmbH, Nürnberg, Germany) with a used L/D ratio of 40D, co-rotating screws, a screw speed of 400 rpm, and a mass throughput of around 8–10 kg/h. Three gravimetric feeders, two Brabender DSR28 for the pellets and one Brabender Minitwin for stabilizer powder (Brabender Technologie GmbH & Co. KG, Duisburg, Germany) were used to ensure a consistent ratio of the virgin material, the recyclate, and a confidential recipe of processing and long-term stabilizers, therefore primary and secondary antioxidants, to ensure no further degradation occurs during the compounding of the materials. Incorporation of stabilizers is an important tool to raise the resistance of the resin and subsequently the pipe against degradation and the effectiveness of primary and secondary antioxidants is well investigated within scientific literature [26], academic theses [27], and the scientific community [28]. Nevertheless, the stabilization of the materials should not influence the mechanical properties measured within the scope of this paper, as no aging was applied to the specimens and no lengthy tests in media and/or at elevated temperatures were conducted. Therefore, the effect of the applied stabilization was not investigated in the present paper.

Since PCRs contain unknown types and dosages of stabilizers, the effect of the PCRs on the degradation stability of resulting compounds would be an interesting topic to investigate in future research.

**Figure 1:**
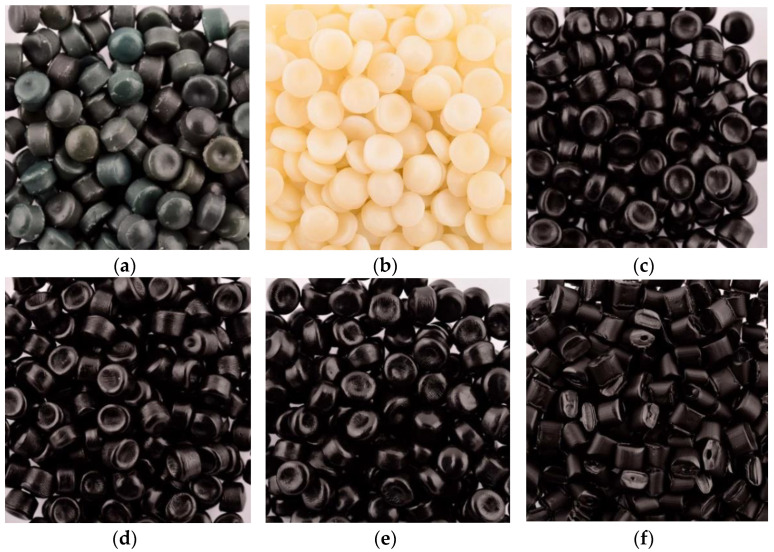
Images of the used pellets of rPE-A (**a**), rPE-B (**b**), PE100-RC (**c**), PE100 (**d**), PE80 (**e**), and representative for all compounds B75 (**f**), as their pellets are visually indistinguishable from each other.

As PE100-RC materials demonstrate the highest slow crack growth (SCG) resistance, compounds containing PE100-RC are expected to deliver the highest possible performance in this area. Therefore, blends containing PE100-RC and 25 m%, 50 m%, and 75 m% recyclate were produced with rPE-A and rPE-B, respectively. Compounds containing PE100-RC and rPE-A are henceforth called A25, A50, and A75. Those containing PE100-RC and rPE-B will be called B25, B50, and B75. A list of all compounds together with the blending ratio is presented in Table 1, and representative images with identical exposure settings of used pellets are shown in Figure 1.

The MFR measurements were conducted at 190 °C under a 5 kg static load on a Zwick/Roell Mflow melt flow indexer (ZwickRoell GmbH & Co. KG, Ulm, Germany) according to ISO 1133-1 [29]. Cuts were made with every 3 mm piston movement. The time between cuts was measured and each extrudate was weighed on an ABS 220-4 electronic balance (Kern & Sohn GmbH, Balingen, Germany). The extra- and interpolation to 10 min calculated the MFR in g/10 min for each cut. For each material, one measurement was conducted. Within one measurement, 6 cuts were made and used for the calculation of average values and standard deviations.

Most of the tested materials show an MFR lower than 1 g/10 min and specimens of it should, according to ISO 17855-2 [30], be produced from pressed sheets. rPE-A and A75 exceed the MFR boundary of 1 g/10 min and thus specimens should be injection-molded. To ensure a high reproducibility of the specimens and maintain a uniform specimen preparation method, all multipurpose specimens (MPS) were produced via injection molding according to ISO 3167 [31] and ISO 17855-2 [30] on an Engel Victory 60 (Engel Austria GmbH, Schwertberg, Austria). Specimens were conditioned at 23 °C and 50% relative humidity for 3–5 days. After conditioning, these specimens were used for tensile testing, and after subsequent cutting to Type 1 specimens and notching, both in accordance with ISO 179-1 [32], also used for Charpy notched impact testing.

Dynamic scanning calorimetry (DSC) measurements were carried out on a PerkinElmer differential scanning calorimeter DSC 8500 (PerkinElmer Inc., Waltham, MA, USA). Samples were cut from shoulders of injection-molded MPS and encapsulated in perforated aluminum pans. The average sample weight was around 8 mg. The procedure consisted of an initial heating phase, subsequent cooling, and a second heating phase, each in the temperature range of 0 °C to 200 °C with a constant heating/cooling rate of 10 K/min with nitrogen as the purge gas and a flow rate of 20 mL/min. The DSC measurements were accomplished to determine the melting peak in the second heating phase, which is characteristic for the semi-crystallinity achieved under controlled cooling in the DSC device. To determine the melting enthalpy, the area of the melting peak was integrated in the temperature ranges from 60 °C to 135 °C for the PE fraction, and from 135 °C to 168 °C for the PP fraction of the materials. Due to the normalization of the heat flux via the specimen mass, the thermograms can be shown as normalized heat flux (W/g) over time (s) and the area of the peak (W/g · s) will calculate normalized melting enthalpy *∆H_m_* (J/g). For each material, five samples, each cut from an individual MPS, were used for the calculation of average values and standard deviations. Measurements were made according to ISO 11357-1 [33] and ISO 11357-3 [34].

None of the cited pipe standards set demands for tensile modulus or yield stress on specimen level. However, some pipe standards demand a certain value of ring stiffness, a component level test on a produced pipe. The standard for district heating pipes EN 253 [4] demands a strain at break of above 350% measured using a specimen with a geometry according to Type 5 specimen from ISO 527-3 [35], which will be punched from a pipe. Nevertheless, for a general comparison of basic mechanical material parameters, tests on specimen level are more suitable and reproducible. Therefore, the tensile properties (tensile modulus, yield stress, and strain at break) were examined with a universal testing machine Zwick/Roell AllroundLine Z020 equipped with a Zwick/Roell multi-extensometer strain measurement system with MPS. Test parameters and MPS were used according to ISO 527-1 [36] and ISO 527-2 [37] with a traverse speed of 1 mm/min for tensile modulus determination until a strain of 0.25%, and after that 50 mm/min until failure. Calculations of tensile modulus, yield stress and strain at break were done in accordance with ISO 527-1 [36]. Therefore, the tensile modulus was calculated as the slope of the stress/strain curve between 0.05% and 0.25% via regression; the yield stress was the stress at the first occurrence of strain increase without a stress increase; and the strain at break was the strain when the specimen broke. The strain was recorded via a multi-extensometer until yield. From there, the nominal strain was calculated via Method B according to ISO 527-1 [36] with the aid of the crosshead displacement. This process is integrated and automated in the used testing software TestXpert III (v1.61, ZwickRoell GmbH & Co. KG, Ulm, Germany). For each material, five MPS were tested for the calculation of average values and standard deviations. The values obtained with virgin pipe grades will act as benchmarks instead of the standards-demanded values.

Impact properties were determined according to ISO 179-1 [32] on a Zwick/Roell HIT25P pendulum impact tester. After pretests to determine the suitable pendulum size (absorbed energy between 10% and 80% of the available energy at impact), a 2 Joule pendulum, the pendulum with the highest available energy that still conforms to these requirements, was chosen for all tests. Notches were produced with a Leica RM2265 microtome (Leica Biosystems Nussloch GmbH, Nussloch, Germany) and measured on an Olympus SZX16 stereomicroscope (Olympus K.K., Tokyo, Japan). Test conditions were 23 °C test temperature with Type 1 specimen, edgewise blow direction, and notch Type A, i.e., a 0.25 mm notch radius, short ISO 179-1/1eA, which is one of the preferred methods of the standard [32]. For each material, ten specimens were tested for the calculation of average values and standard deviations.

**Figure 2:**
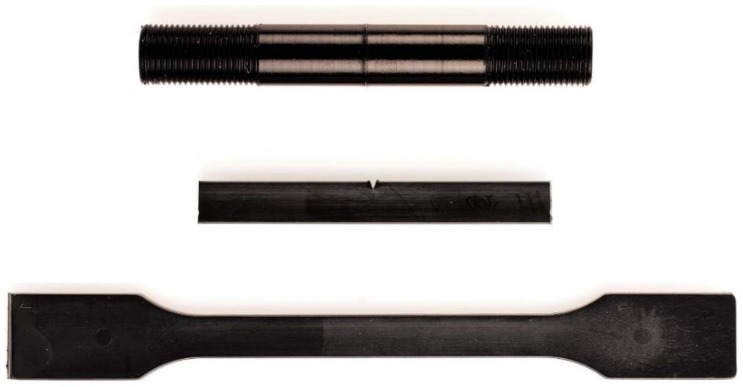
Image of the specimens used in this work made from B75 from top down: CRB specimen lathed from a pressed plate, Type 1 Charpy specimen cut from an MPS, and injection-molded MPS.

For the investigation of long-term SCG resistance, fatigue crack growth (FCG) experiments, which measures the SCG resistance under cyclic loading, with cracked round bar (CRB) specimens following ISO 18489 [13], were conducted. For specimen production, plates of size 16 mm × 120 mm × 150 mm were compression-molded in a specifically designed positive mold with the help of a hydraulic press from the Langzauner Perfect line (Langzauner GmbH, Lambrechten, Austria). Within the fully automated program, 280 g pellets were heated within the mold from room temperature to 180 °C with the weight of the mold on top of the material. An integrated temperature sensor allows for direct measurement of the mold temperature and when the internal temperature of 180 °C was reached, it was held for 15 min. After that, slow cooling with a cooling rate of 2 K/min was started. Depending on the viscosity of the specimen, the full pressure of 10 MPa was applied to the material upon reaching a temperature between 135 °C to 155 °C. Applying full pressure at higher temperatures leads to too much melt displacement. After reaching 40 °C, the pressure was released, the mold opened, and the plate manually removed. The produced plates were conditioned at 23 °C and 50% relative humidity for at least three days before being cut into bars and lathed on an EMCO turning lathe (EMCO GmbH, Hallein, Austria) to CRB specimens according to ISO 18489 [13]. Therefore, round specimens with a diameter of 14 mm and a circumferential notch with the depth of 1.5 mm (resulting in a ligament diameter of 11 mm) and M14 × 1.25 threads on both sides for clamping were produced. A 0.3 mm thick industrial-grade razor blade was used to notch the specimen. Before testing, the specimens were conditioned at 23 °C and 50% relative humidity for another day after being notched. The CRB specimens were tested with an electro-dynamic testing machine of the type Instron ElectroPuls E10000 (Illinois Tool Works Inc., Glenview, IL, USA). Sinusoidal loading profiles with a frequency of 10 Hz, an R-ratio of 0.1 and individually adjusted initial stress intensity factor ranges (Δ*K_ini_*) were used to achieve testing times between 10 h and 100 h. An in situ optical measurement of the crack length over the whole circumference of the specimen was used for investigations of crack growth [23]. Characteristic double logarithmic FCG kinetic curves were plotted to provide the relationship between the FCG rate, *da/dN* in mm/cycle, and the stress intensity factor range, Δ*K_I_* in MPa·m^0.5^. Per material, at least one specimen was tested. An image of a ready-for-testing CRB specimen [13], together with a Charpy impact testing Type 1 specimen [32], and an MPS [31], is shown in Figure 2.

## 3. Results

### 3.1. Melt Flow Rate

The melt flow rate is an important indicator of the rheological behavior of the material and in most pipe standards the first property to fulfill. Although most standards agree on a minimum MFR of 0.2 g/10 min, the maximum tolerated MFR differs. Within the non-pressure pipe applications, the allowed maximum MFRs range from up to 1 g/10 min for the PE-HD outer layer of district heating pipes [4] to up to 1.6 g/10 min for non-pressure underground structured-wall drainage and sewerage pipes [38,39]. The values should be measured according to ISO 1133 at 190 °C and with 5 kg. These values apply to both pipes and fittings.

**Figure 3:**
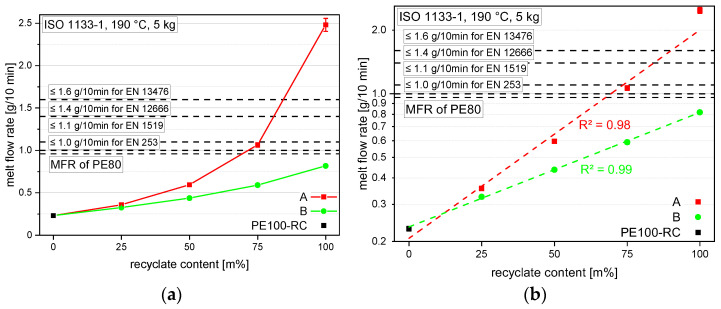
Graphical illustration of MFR values of the virgin material PE100-RC (as 0 m% recyclate content data point for both compounding series) both recyclates (at 100 m% recyclate content) and the compounds containing 25 m%, 50 m%, and 75 m% rPE-A and rPE-B, respectively. The data is plotted linearly (**a**) and logarithmically (**b**) to emphasize the logarithmic trend. The vertical bars show the sample standard deviation.

Despite their mentioned data within the data sheets, all virgin materials and recyclates were measured together with the compounds for an accurate comparison, and results are shown in Figure 3. The used PE100-RC (shown at a recyclate content of 0 m%) shows the lowest value with 0.23 g/10 min and rPE-A the highest value with 2.48 g/10 min. rPE-B already has an applicable MFR of 0.82 g/10 min for every non-pressure pipe standard, both shown at a recyclate content of 100 m%. Although the MFR values of both compounding series show non-linear declines with lower recyclate contents in the linear MFR diagram (Figure 3a), almost linear trends (*R²* values of 0.98 and 0.99) can be confirmed when plotting the MFR values logarithmic, as seen in Figure 3b.

Although rPE-A starts with too high an MFR to be used in any of the discussed pipe standards, by adding 25 m% PE100-RC, the MFR drops to a value of 1.07 g/10 min, which is already useful for three of the four standards discussed. By adding another 25 m% PE100-RC, the resulting A50 compound shows an MFR of 0.6 g/10 min and is therefore appropriate for all four standards. The compound with the lowest recyclate content of only 25 m%, and therefore with 75 m% PE100-RC, shows an even lower MFR of 0.36 g/10 min. rPE-B, on the other hand, starts with a low enough MFR of 0.82 g/10 min to be used without further compounding in all four discussed standards. By adding PE100-RC, the MFRs decrease to 0.59 g/10 min, 0.44 g/10 min, and lastly 0.33 g/10 min, staying below the MFR values of the corresponding rPE-A compounds with the same recyclate content, although with a decreasing difference.

### 3.2. Dynamic Scanning Calorimetry

**Figure 4:**
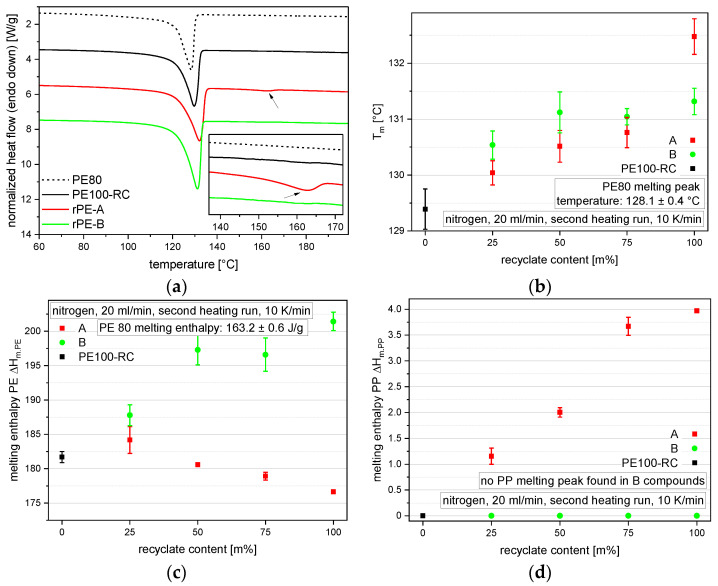
Graphical illustration of DSC results. Thermograms of the virgin materials and the pure recyclates are shown in (**a**). Results of all materials are shown in terms of PE melting temperatures (**b**), PE melting enthalpies (**c**), and PP melting enthalpies (**d**). The vertical bars show the sample standard deviation.

The DSC measurements are used to assess the crystallinity of the materials and detect foreign polymers. It is known that contaminations, either by other polymers or residues from the recycling process or the application, can affect the performance of polyolefins [40]. The thermograms of the pure substances show clear PE melting peaks at around 130 °C for all materials, as seen in Figure 4a. Only the thermogram of rPE-A reveals a measurable endothermal peak (indicated by the arrows) at 160.4 °C, representing a PP melting peak. The PE melting peaks differ in PE melting peak temperature *T_m_* (Figure 4b) and PE melt enthalpy Δ*H_m.PE_* (Figure 4c). Both recyclates offer higher PE melting peak temperatures (*T_m_* for rPE-A: 132.5 °C and rPE-B: 131.3 °C) than both virgin pipe materials (*T_m_* for PE100-RC: 129.4 °C and PE80: 128.1 °C). The melting peaks of the compounds range, as expected, between the two blending partners. The melting enthalpy of the recyclates differ. Although rPE-B offers a higher Δ*H_m.PE_* of 201.42 J/g than PE100-RC with Δ*H_m.PE_* of 181.7 J/g, rPE-A offers a lower Δ*H_m.PE_* of 176.6 J/g. The virgin PE80 material shows the lowest melting enthalpy of 163.2 J/g. For most compounds, the melting enthalpy values again lie in a linear relationship with the recyclate content between the two blending partners. However, A25 shows a higher melting enthalpy than anticipated, but could be explained with nucleation by foreign particles by compounding and the higher standard deviation of the measurement. These enthalpy values would correspond in pure PE materials to crystallinities *w_c_* of 55.7% for the PE80 grade, 60.3% for rPE-A, 62% for PE100-RC, and 68.8% for rPE-B [41]. The PP melting peak, which can be seen in the thermogram of rPE-A in Figure 4a, can also be measured in all A compounds, as seen in Figure 4d. A steady decrease of PP melting enthalpy Δ*H_m.PP_* can be observed, starting at 4.0 J/g for rPE-A, 3.7 J/g for A75, 2.0 J/g for A50, and 1.2 J/g for A25. Due to the high variation in crystallinity found within different PP homopolymers and PP copolymers, no reliable estimate for the PP content in rPE-A can be made.

### 3.3. Tensile Properties

**Figure 5:**
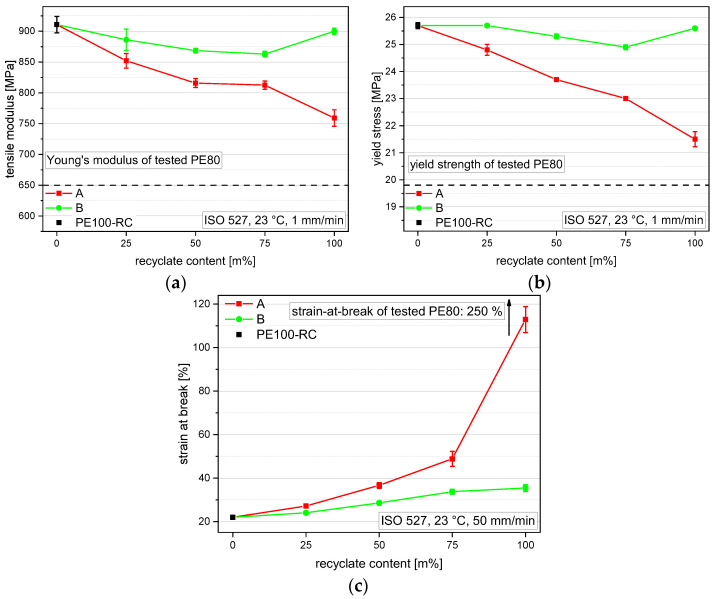
Graphical illustration of tensile modulus (**a**), yield stress (**b**), and strain at break values (**c**) of the virgin material PE100-RC (as 0 m% recyclate content data point for both compounding series), both recyclates (at 100 m% recyclate content), and the compounds containing 25 m%, 50 m%, and 75 m% rPE-A and rPE-B, respectively. The vertical bars show the sample standard deviation.

PE100-RC shows the highest tensile modulus of around 911 MPa and the PE80 grade shows the lowest tensile modulus of 650 MPa, as seen in Figure 5a. The recyclates lie in between but differ significantly with rPE-A at 759 MPa and rPE-B at 900 MPa. The A compounds show decreasing moduli with rising recyclate content, and all values lie in between the values of the respective blending partners PE100-RC and rPE-A with A25 at 852 MPa, A50 at 816 MPa, and A75 at 813 MPa. The B compounds show a different trend. Although the compounds also decrease in tensile modulus with increasing recyclate content, all compounds show a lower tensile modulus than both blending partners, with B25 at 886 MPa, B50 at 869 MPa, and B75 at 863 MPa. This seems to be an antagonistic effect of mixing the blending partners together and was also found for the flexural modulus of one of the blending series within the study of Juan et al. [14].

Comparable trends can be found with the yield stress values, shown in Figure 5b. The A compounds show a linear decrease with added recyclate content between the values of PE100-RC at 25.7 MPa and rPE-A at 21.5 MPa with A25 at 24.8 MPa, A50 at 23.7 MPa, and A75 at 23.0 MPa. The pure rPE-B shows a higher 25.6 MPa than B50 at 25.3 MPa and B75 at 24.9 MPa, therefore showing a similar antagonistic effect as for the tensile modulus. Only B25 shows a higher yield stress than rPE-B with 25.7 MPa.

The strain at break values, as seen in Figure 5c, show trends similar to MFR. Pipe materials usually achieve much higher strain at break values, but only when they are produced from pressed sheets. When low-MFR materials are injection-molded, the achievable strain at break is highly influenced by its rheological behavior. Since the shear stress during injection molding decreases with increasing flowability, the polymer chains are less oriented and allow for a higher deformation. Nevertheless, the strain at break performance of recyclates highly depends on contaminations [40]. This also explains the high strain at break value of PE80 (250%) at comparable MFR to rPE-B and lower MFR compared to rPE-A.

### 3.4. Charpy Notched Impact Strength

**Figure 6:**
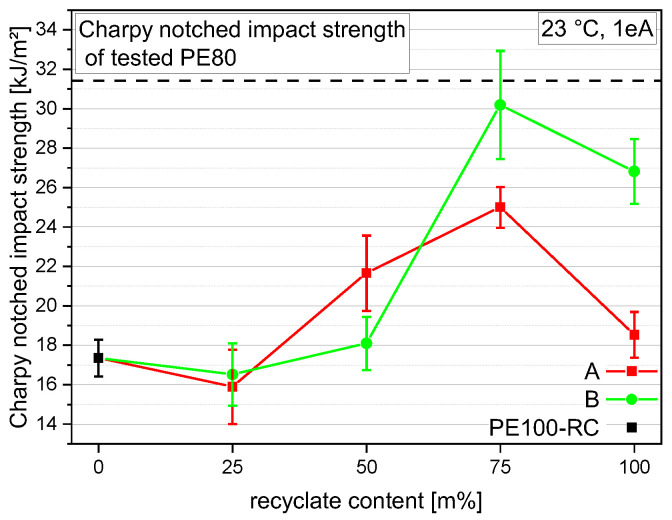
Graphical illustration of Charpy notched impact strength of the virgin material PE100-RC (as 0 m% recyclate content data point for both compounding series), both recyclates (at 100 m% recyclate content), and the compounds containing 25 m%, 50 m%, and 75 m% rPE-A and rPE-B, respectively. The vertical bars show the sample standard deviation.

The Charpy notched impact strength of the compounds of all materials are depicted in Figure 6. Although PE100-RC shows a very low value of 17.3 kJ/m², PE80 shows the highest value at 31.4 kJ/m². In both cases, the addition of 25 m% recyclate to the PE100-RC lowers the performance, resulting in 15.9 kJ/m² for A25 and 16.5 kJ/m² for B25. The other way around, adding 25 m% PE100-RC to the recyclate had a beneficial effect on both recyclates. rPE-A, starting at 18.5 kJ/m², rises to 25 kJ/m² for A75 and the already high Charpy notched impact strength of rPE-B with 26.8 kJ/m² goes up to 30.2 kJ/m² for B75. The compounds with 50 m% recyclates show intermediate values of 21.7 kJ/m² for A50 and 18.1 kJ/m² for B50.

### 3.5. Fatigue Crack Growth Resistance

**Figure 7:**
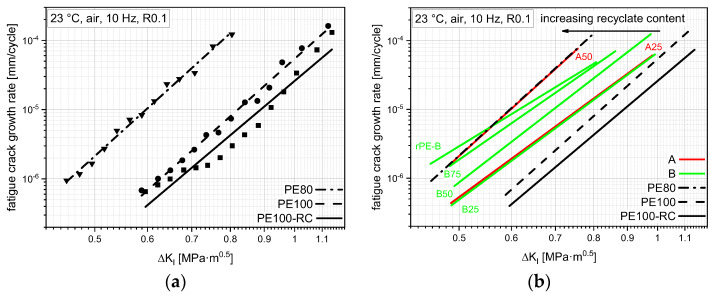
FCG kinetic curves of the three virgin benchmarks (**a**) and of all tested materials including compounds (**b**). rPE-A and A75 did not show any measurable crack growth until failure and therefore are not depicted.

FCG experiments on CRB specimens from the virgin materials show a broad range of FCG resistances, as can be seen in Figure 7a. The measurement points depict the FCG rates over stress intensity factor range values Δ*K_I_* during the measurement of each specimen. The Δ*K_I_* value depends on the geometry, applied force range, and crack length as can be seen in the following Formulas (1)–(3) developed by Benthem and Koiter [42] and used within the CRB test standard ISO 18489 [13].

(1) $$\Delta K_{I}=\frac{\Delta F}{\pi \cdot b^{2}}\cdot \sqrt{\frac{\pi \cdot a\cdot b}{r}}\cdot f\left(\frac{b}{r}\right)$$

(2) b=r−a

(3) $$f\left(\frac{b}{r}\right)=\frac{1}{2}\cdot \left[1+\frac{1}{2}\cdot \left(\frac{b}{r}\right)+\frac{3}{8}\cdot {\left(\frac{b}{r}\right)}^{2}-0.363\cdot {\left(\frac{b}{r}\right)}^{3}+0.731\cdot {\left(\frac{b}{r}\right)}^{4}\right]$$

where Δ*K_I_* is the stress intensity factor range in loading Mode I [43], Δ*F* the applied force range, *a* the crack length, *r* the radius of the specimen, *b* the ligament (*r–a*), and *f(b/r)* a geometry function.

As the crack length is the only changing parameter during the measurement, the change in Δ*K_I_* can also be seen as a progression from the starting crack length of around 1.5 mm to fracture at typically 3–4 mm crack length. The only parameter changed between material tests was the maximum force, and hence, calculated with the force ratio *R* of 0.1, the force ranges to accommodate the different SCG resistances and maintain economical testing times. The resulting trend in rising Δ*K_I_* with rising FCG rate is called FCG kinetics curve. FCG kinetic curves which lie at higher Δ*K_I_* values and/or lower FCG rates represent a better FCG resistance.

PE100-RC was tested at higher Δ*K_I_* values compared to PE80, but shows comparable FCG rates, as seen in Figure 7a. At overlapping Δ*K_I_* values, e.g., 0.7 MPa·m^0.5^, PE100-RC shows a 27 times lower crack growth rate compared to PE80. Linear fits from these measurement points seen in Figure 7a are made to raise the visibility of the FCG trends.

FCG kinetic curves of all measurable materials are presented in Figure 7b. No FCG data are shown for A75 and rPE-A, as the specimen failed without measurable crack growth, even at low loadings. The other compounds show clear trends of falling FCG resistance with rising recyclate content. A50 shows a very comparable FCG kinetic curve to PE80 and B75 intersects PE80 at lower Δ*K_I_* values, inducing a worse FCG resistance at the more lifetime-determining lower loads [22]. A25, B25, and B50 show better FCG resistances than PE80 over the whole test range.

## 4. Discussion

**Figure 8:**
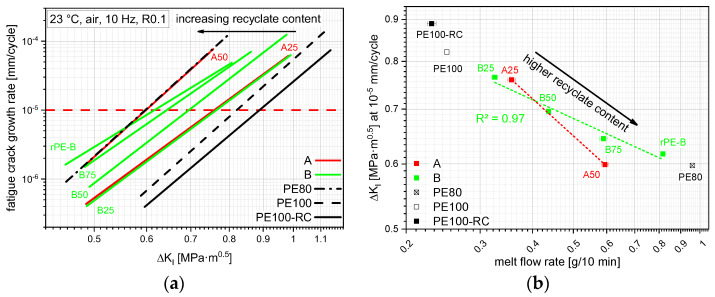
FCG kinetic curves of all tested materials (**a**) with a horizontal red line depicting the predefined FCG range to obtain stress intensity factor ranges for a correlation with the MFR values (**b**). The horizontal bars show the sample standard deviation.

A way of quantifying FCG performance is to compare them at a similar FCG rate. When comparing the FCG kinetics of all tested materials at an FCG rate of 10^−5^ mm/cycle, as depicted by the red dashed line in Figure 8a and plotting the resulting stress intensity factor range (Δ*K_I_*) values over their respective MFR, an interesting correlation can be devised as shown in Figure 8b. As previously shown in Figure 3, the MFR rises with rising recyclate content, while the FCG resistance lowers with rising recyclate content, as seen before. Although there is a significant drop in performance by adding as little as 25 m% recyclate, the B compounds, together with the used rPE-B, show an almost linear correlation with an R² of 0.97. Although A25 and B25 show comparable results, adding more rPE-A to the compounds lowers the FCG resistance significantly more than adding the same amount of rPE-B. This is foremost depicted by the different MFRs at 50 m% recyclate content where B50 provides lower 0.43 g/10 min compared to A50 with 0.6 g/10 min, but also by the gap in Δ*K_I_* (0.7 MPa·m^0.5^ vs. 0.6 MPa·m^0.5^). However, even at comparable MFR values (0.59 g/10 min vs. 0.60 g/10 min), B75 shows a higher SCG resistance than A50. This performance difference at comparable MFRs can be attributed to a detrimental influence of the PP contamination within rPE-A as shown before in Figure 4d. Although the difference in Δ*K_I_* values is not that big (0.64 MPa·m^0.5^ to 0.60 MPa·m^0.5^), the FCG rate at this Δ*K_I_* value, as shown in Figure 8a, differs by a factor of 1.6. Due to the different slopes between A50 and B75 within Figure 8a, this factor ranges between 1.0 and 2.6, depending on the Δ*K_I_* value.

**Figure 9:**
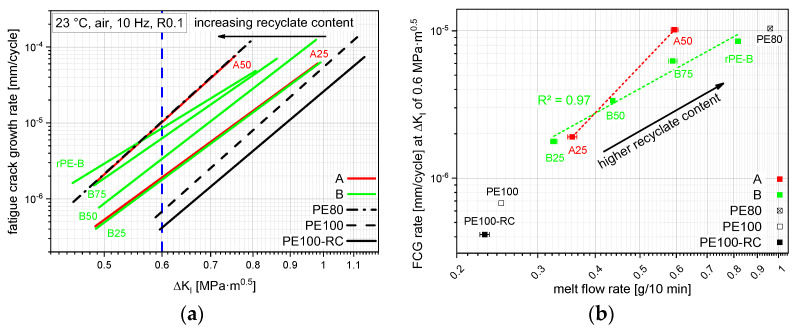
FCG kinetic curves of all tested materials (**a**) with a vertical blue line depicting the predefined stress intensity factor range to obtain FCG rates for a correlation with the MFR values (**b**). The horizontal bars show the sample standard deviation.

Another way of quantifying the FCG resistance is the comparison of FCG rates at the same stress intensity factor range. A comparison of FCG rates of the different materials at the same Δ*K_I_* of 0.6 MPa·m^0.5^, depicted by the blue dashed line in Figure 9a, is thus also possible and shown in Figure 9b. The trends and the *R²* of the B compound correlation are similar to the ones shown in Figure 8b, though the differences in numbers are bigger.

Several publications show SCG behavior of recycled [16] or non-virgin PE [44], respectively, or the effect of cross-contamination [45] which can be a problem with using recycled plastics. Only two previous publications have blended post-use recyclates with pipe-grade PE and investigated their SCG behavior [14,15]. These two publications show a gradual decrease in SCG resistance with rising recyclate content. More importantly, a decrease in SCG resistance with decreasing angular frequency of the cross-over point in a rheological measurement [15] or decreasing mass-average molecular weight [14] was shown, which agrees with the findings in this paper concerning the correlation of FCG performance with the MFR. No direct comparisons between our results and the results of these publications can be made, as they used different methods. However, Juan et al. [14] also compares his findings to general failure time areas for PE100- and PE80-grade materials. Based on their measurements, up to 35% of the used blow-molding recyclate can be incorporated into a PE100 compound to achieve PE80 SCG resistance. Considering the raised SCG resistance of PE100-RC which was used in the present paper, the achieved higher possible recyclate contents are plausible.

## 5. Conclusions

Polyethylene (PE) recyclates from packaging waste streams usually have higher melt flow rates (MFR) and lower resistance against slow crack growth (SCG), both properties that are relevant for pipe production. The blending of these recyclates with low-MFR, SCG-resistant PE100-RC leads to compounds which compete with a virgin PE80 injection-molding grade. Although the SCG resistance of virgin pipe grades is highly optimized and dependent on chemical and morphological factors [46], the SCG resistance of the compounds tested in this paper with rPE is mostly described by its MFR. However, the MFR determines not only the SCG resistance of the compounds, as is shown by compounds with similar MFRs, but also different SCG resistances. Other influences such as contaminations can also attribute to performance differences within the compounds. The authors want to state that while the SCG resistance presents a determining factor for pipe lifetime, many other properties are necessary for successful pipe production and admission as pipe resin.

## Figures and Tables

**Table 1:** List of compounds and their respective amounts of blending partners.

	PE100-RC	rPE-A	rPE-B
	m%	m%	m%
A25	75	25	-
A50	50	50	-
A75	25	75	-
B25	75	-	25
B50	50	-	50
B75	25	-	75
